# Bioactive Guided Fractions of *Annona reticulata* L. bark: Protection against Liver Toxicity and Inflammation through Inhibiting Oxidative Stress and Proinflammatory Cytokines

**DOI:** 10.3389/fphar.2016.00168

**Published:** 2016-06-22

**Authors:** Raghuram Kandimalla, Suvakanta Dash, Sanjeeb Kalita, Bhaswati Choudhury, Sandeep Malampati, Kasturi Kalita, Jibon Kotoky

**Affiliations:** ^1^Drug Discovery Laboratory, Life Sciences, Institute of Advanced Study in Science and TechnologyGuwahati, India; ^2^Girijananda Chowdhury Institute of Pharmaceutical ScienceGuwahati, India; ^3^School of Chinese Medicine, Hong Kong Baptist UniversityHong Kong, China; ^4^Department of Pathology, Hayat HospitalGuwahati, India

**Keywords:** antioxidant, liver toxicity, cytokines, inflammation, oxidative stress

## Abstract

Herbal medicine is popularized worldwide due to its ability to cure the diseases with lesser or no side effects. North Eastern part of India comes under one of the world biodiversity hotspots which is very rich in traditional herbal medicine. *Annona reticulata* L. (Annonaceae) is one such plant used for the treatment of inflammatory diseases, liver ailments and diabetes by traditional healers. The present study was aimed to scientifically validate this folk knowledge and to develop an herbal remedy through evaluating bioactive guided fractions of *A. reticulata* (AR) bark against hepatotoxicity and inflammation using *in vitro* and *in vivo* models. Results of this study demonstrates that among all fractions of AR bark, methanol extract and its water fraction possess strong anti-oxidant ability and showed protection against CCl_4_ induced toxicity in HepG2 cell lines and rats. Both the fractions also exhibit dose dependent anti-inflammatory activity against carrageenan induced inflammation in rats. Water fraction showed potent response in the entire tests conducted than methanol extract, which states that polar components of the AR bark methanol extract were responsible for these activities. Further, from the experiments conducted to elucidate the mechanism of action, the results revealed that AR bark showed liver protection and anti-inflammatory response through inhibiting the oxidative stress and inflammatory cytokines.

## Introduction

Reactive oxygen species (ROS) cause oxidative damage and lipid peroxidation in cells which can potentially leads to different diseases like cancer, inflammation, aging, heart problem, and severe liver damage (Ha et al., 2010; Chen et al., 2011). Liver is the second largest organ in the body which involves in vital functions like cleansing blood, vitamin synthesis, regulation of supply of body fuel, cholesterol regulation, balancing hormone regulation, and drug metabolism. Different ailments like virus, chemicals, and chronic alcoholism cause damage to the liver by producing vast number of ROS (Hou et al., 2013). More than 600 chemical substances can cause liver injury, among all carbon tetra chloride (CCl_4_), paracetamol and ethanol are more predominant (Cengiz et al., 2013). Inflammation caused due to tissue damage through different stimuli like irritants and pathogen or due to physical injury. There are very few drugs available in the market for the treatment of liver ailments and inflammation with fewer side effects despite of its pharmacological action. In this regard, it is necessary to develop novel drug candidates with less or no side effects (Huang et al., 2012). Ayurvedic medicine is so popular worldwide because of its effective ness and lesser toxicity. Medicinal plants are the tremendous source of antioxidants and phytochemicals which have the ability to treat liver aliments and inflammation (Feng et al., 2011; Pareek et al., 2013). To decrease the side effects by the synthetic drugs in the market, scientists are now focusing on developing the herbal based remedy (Kuete and Efferth, 2010).

Antioxidant ability of the plant components can help to fight against different disease conditions like organ damage; inflammation and cancer etc., through ROS neutralization inside the body. Natural antioxidants are safer and healthy than synthetic antioxidants used in food materials (Su et al., 2009). *Annona reticulata* L. (Annonaceae) is a small tree commonly called as Ramphal, Bullock’s heart and custard apple, native to India, West Indies and tropical America, mainly cultivated for fruit production. Traditionally this plant is used as antiparasitic, insecticide, antidiarrheic, and antidysenteric. Different plant parts of *A. reticulata* have been reported for anti-hyperglycemic, analgesic, cytotoxic, anti-prolifiratory, and CNS depressant activities (Chavan et al., 2014; Jamkhande and Wattamwar, 2015). This plant is a good source of different bioactive phytochemicals like acetogenins (Chang et al., 1998), cycloreticulins A and B, cyclooctapeptides, cycloreticulin C, glabrin A, cyclopeptides (Wele et al., 2009; Jamkhande et al., 2016). Stem bark of this plant contains different chemical constituents like dopamine, salsolinol, coclaurine, 16-a-hydroxy-(e)-kauran-19-oic acid, diterpenes (e)-kaur-16-en-19-oic acid, reticullacinone, methyl-17-hydroxy-16-b-(e)-kauran-19-oate, rolliniastatin and molvizarin (Hisham et al., 1994; Nirmal et al., 2010; Bhalke and Chavan, 2011). A substantial amount of population of Assam, Arunachal Pradesh, and Nagaland of India are using bark of this plant to cure different liver ailments, inflammation and diabetes. To scientifically validate this claim and to develop an herbal drug remedy the present study aims to investigate the bioactive guided fractions of *A. reticulata* bark for antioxidant, hepatoprotective and anti-inflammatory activities using both *in vitro* and *in vivo* models.

## Materials and Methods

### Chemical and Drugs

Cell culture media and related chemical obtained from Invitrogen, Life Technologies, USA. ELISA kits procured from R&D Systems, USA. Biochemical kits purchased from Accurex Biomedical, Pvt. Ltd., Mumbai. All the other chemicals were analytical grade and obtained from Sigma-Aldrich, Co., St. Louis, MO, USA.

### Plant Collection and Identification

*Annona reticulata* bark was collected from Kamrup district (26.3333° N, 91.2500° E), Assam in the month of January, 2015. Plant was identified by a taxonomist at North East India Ayurvedic Institute, Guwahati. A voucher specimen number (1801/IASST/2014-15) was deposited at herbarium, Drug Discovery Laboratory, Institute of Advanced Study in Science and Technology for future reference.

### Extraction and Bioactive Guided Fractionation

**FIGURE 1:**
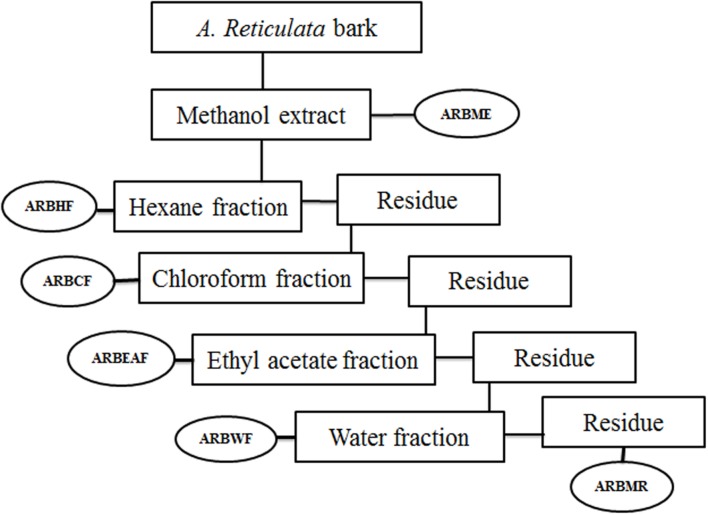
**Schematic representation of bioactive guided fractionation of *Annona reticulata* bark**. ARBME, *A. reticulata* bark methanol extract; ARBHF, *A. reticulata* bark hexane fraction; ARBCF, *A. reticulata* bark chloroform fraction; ARBEAF, *A. reticulata* bark ethyl acetate fraction; ARBWF, *A. reticulata* bark water fraction; ARBMR, *A. reticulata* bark methanol residue.

*Annona reticulata* bark (ARB) was dried at room temperature (25–27°C) and grinded into a coarse powder. 10 kg of the dried powder was subjected to simple maceration with methanol for 72 h. The *A. reticulata* bark methanol extract (ARBME) was concentrated under pressure using rota evaporator (Buchi, Switzerland) to yield dry residue of 500 gm. A part of methanol extract was subjected to gradual fractionation using different solvents of increasing polarity viz, hexane (ARBHF), chloroform (ARBCF), ethyl acetate (ARBEAF), and water (ARBWF). Briefly, 400 gm of ARBME was suspended into one liter of hexane and stirred vigorously by using magnetic stirrer for 24 h at room temperature. Further the hexane fraction was collected through filtration and the undissolve residue was collected and dried to continue the further fractionation. After extracting with all the solvents, the remaining methanol residue was collected and dried (ARBMR). The schematic representation of bioactive guided fraction is shown in **Figure 1**. Main mother methanol extract and its five fractions were prepared for testing biological activity and stored in 4°C. All the samples were tested within 3 months of extraction and thawed before use.

### *In Vitro* Antioxidant Activity

#### DPPH Radical Scavenging Assay

Diphenyl picryl hydrazine (DPPH) assay is widely used method to determine the ability of phytochemicals to neutralize the free radicals (Choudhury B. et al., 2016; Kalita H. et al., 2016). Briefly, 2.7 mL of 0.2 mM DPPH solution was added to 0.3 mL of the plant extracts/fractions at various concentrations. The reaction mixture was vigorously shaken and incubated at room temperature for 1 h; absorbance was measured at 517 nm. The radical scavenging activity was calculated as follows: scavenging rate = [(As - Ai)/As] × 100, where As is the absorbance of pure DPPH and Ai is the absorbance of DPPH in the presence of various extracts. Ascorbic acid at different concentrations identical to the experimental samples was used as references.

#### Reducing Power Assay

The reducing power of the extracts was estimated by following (Oyaizu, 1986). Increased concentrations of 0.2 mL extracts were mixed with 2.5 mL of phosphate buffer (0.2 M, pH 6.6) and 2.5 mL of potassium ferricyanide (1%). After incubation at 50°C for 20 min, 2.5 mL of trichloroacetic acid (10%) was added, and each mixture was centrifuged at 1000 rpm for 10 min. Then, 2.5 mL of the supernatant was collected and mixed with 2.5 mL of deionized water and 0.5 mL of ferric chloride (0.1%). The absorbance was measured at 700 nm. The increased absorbance of the reaction mixture indicated increased reducing power. BHT was used as standards for comparison.

#### Total Antioxidant Activity

Measurement of total antioxidant capacity of plant extracts were performed by photochemiluminescence method in the Photochem instrument, Germany. The lipophilic and hydrophilic antioxidants were measured with the kits commercially available from Photochem, Germany. Total antioxidant activity of plant extracts was expressed in trolox equivalents for lipophilic antioxidants and ascorbic acid equivalents for hydrophilic antioxidants.

### Cell Culture

Human liver hepatoma cells (HepG2) were obtained from National Center for Cell Sciences (NCCS), Pune, India. The cells were seeded 1 × 10^5^ cells/T_25_ flasks and cultured in DMEM containing low glucose with 10% FBS and Penstrap (antibiotic solution) in CO_2_ Incubator at 37°C. Stock culture was grown in 25 cm^2^ culture flasks and all experiments were done in 96 well plates (Life Technologies, USA).

#### *In vitro* Cytotoxicity Assay

Tetrazolium salt assay (MTT) was used to determine the cytotoxic concentration of plant fractions by following the method of Kalita S. et al. (2015, 2016) and Kandimalla et al. (2016). Briefly, the cells from the culture flask was trypsinized and seeded in 96-well plate at 5 × 10^3^ cells/well and incubated in CO_2_ incubator at 37°C for 24 h. After 24 h culture medium was replaced with new medium and ARBME, ARBWF (5, 25, 50,125, 250, and 500 μg/ml) at different concentrations. After 72 h of incubation in CO_2_ incubator at 37°C, medium was removed and 20 μl of 4 mg/ml MTT (pH 7.4) was added in each well. Plate was incubated for 4 more hours and the supernatant was removed and 100 μl of DMSO was added to each well and incubated for 30 min to dissolve the formed formazan. Absorbance was read at 570 nm by using micro plate reader (ThermoFisher, USA). The percentage growth inhibition was calculated by using the following formula:

$$\%\;Growth\,inhibition=100-[\frac{Mean\,\;OD\,\;of\,\;individual\,\;test\,\;group}{Mean\,\;OD\,\;of\,\;control\,\;group}]\times 100$$

#### Protective Effect of *A. reticulata* Fractions in CCl4 Induced Toxicity in HepG2 Cell Lines

HepG2 cells at 1 × 10^5^ cells/ml were adjusted with DMEM medium containing 10% fetal bovine serum. To each well of 96 well microtiter plate 0.1 ml of diluted cell suspension was added and incubated at 37°C for 24 h in CO_2_ incubator. After 24 h of incubation the medium was discarded and expose the cells with different treatments they are as following:

**Group-I (Control):** *Normal control:* Cells were treated with 100 μl of serum free culture medium for 24 h.

*DMSO control:* Cells were treated with 100 μl of serum free culture medium containing DMSO (0.25% v/v) for 24 h.

Silymarin Control: Cells were treated with 100 μl of serum free culture medium containing silymarin (200 μg/ml) for 24 h.

*ARBME Control:* Cells were treated with 100 μl of serum free culture medium containing ARBME (200 μg/ml) for 24 h.

ARBWF control: Cells were treated with 100 μl of serum free culture medium containing ARBWF (200 μg/ml) for 24 h.

**Group-II (CCl4 treatment):** Cells were treated with 100 μl of serum free culture medium containing 1.0% (v/v) CCl4 for 24 h.

**Group-III (Standard treatment):** Cells were treated with 100 μl of serum free culture medium containing 1.0% (v/v) CCl4 and silymarin at different concentrations (50, 100, or 200 μg/m) for 24 h.

**Group-IV (ARBME treatment):** Cells were treated with 100 μl of serum free culture medium containing 1.0% (v/v) CCl4 and ARBME at concentration of (50, 100, or 200 μg/m) for 24 h.

**Group-V (ARBWF treatment):** Cells were treated with 100 μl of serum free culture medium containing 1.0% (v/v) CCl4 and ARBWF at concentration of (50, 100, or 200 μg/m) for 24 h.

#### Cell Viability Assay

Trypan blue exclusion assay (Rambabu and Vijayakumar, 2014) was performed to determine the cell viability. Briefly, after the exposure of cells to different treatment the cells from the wells were trypsinized and centrifuged at 1000 *g* for 10 min at 40°C. The pellet was resuspended in 1 ml ice cold PBS, then 0.1 ml of cell suspension was mixed with 0.1 ml of 0.2% trypan blue. Viable cells were counted using hemocytometer under lite microscope. Percentage of cell viability was calculated using the following formula-

$$\%viability=\frac{\left(Total\,\;number\,\;of\,\;cells-Trypan\,\;blue\,\;stained\,\;cells\right)}{Total\,\;number\,\;of\,\;cells}\times 100$$

#### Measurement of LDH

After the drug treatment, HepG2 cells culture media from all the treatment groups were centrifuged at 2000 rpm for 15 min and supernatant was collected to measure the LDH by Ecoline diagnostic kit. One unit of LDH activity is describe as the amount of enzyme that catalyzes the conversion of lactate to pyruvate to produce 1.0 μmol of Nicotinamide adenine dinucleotide (NADH) per minute.

#### Acute Toxicity Studies

Acute oral toxicity studies were performed according to OECD guidelines to test chemicals. Swiss albino mice of either sex (five animals) was randomly selected were used for this study. Animal was kept overnight fasting with free access to water but not food, next day morning single dose of methanolic extract and active fraction of *A. reticulata* bark at 2000 mg/kg body weight were administered orally to three animals each. Animals were observed for 14 days, if mortality was observed in two out of three animals, then the dose was identified as toxic dose. If mortality was observed in one animal, experiment was repeat again with same dose to confirm the toxic dose. If mortality observed again experiment was continued with low doses (300, 50, and 5 mg/kg body weight).

#### Animals

Adult male rats of Wistar strain weighing 150–200 g (Main experiment) and Swiss albino mice weighing 25–30 g (Acute toxicity studies) were obtained from the Institute of Advanced study in Science and Technology (IASST), Guwahati (India). Animals were maintained at 24°C ± 1°C, with relative humidity of 45–55% and 12:12 h dark/light cycle and had free access to standard pellet diet (Provimi Animal Nutrition, Pvt. Ltd., India) and water throughout the experimental protocol. All experiments were carried out between 09:00 and 17:00 h. The experimental protocol was approved by the Institutional Animal Ethics Committee (IAEC) of IASST, Guwahati (IASST/IAEC/2014-15/746) and performed in accordance to the guidelines of Committee for Control and Supervision of Experimentation on Animals (CPCSEA).

### Effect of *A. reticulata* Active Fraction against CCl_4_ Induced Hepatotoxicity

#### Experimental Design

Adult male Wistar rats Total of 42 animals were randomly divided into seven groups of six animals in each group. All the drug treatment was continued for 14 days and on 14th day single dose of CCl4 1.5 mg/kg, in 1:1 dilution with olive oil was given in i.p. route.

**Group I:** Control animals (D.W for 14 days orally).

**Group II:** Toxic control (0.3% CMC for 14 days orally).

**Group III:** Standard group (Silymarin 100 mg/kg in 0.3% CMC for 14 days orally).

**Groups IV and V:** Treatment groups ARBME at 200 and 400 mg/kg in 0.3% CMC for 14 days orally).

**Groups VI and VII:** Treatment groups ARBWF at 50 and 100 mg/kg in 0.3% CMC for 14 days orally).

After 48 h of CCl4 administration all the animals was sacrificed and blood and liver was collected for biochemical estimation and histopathology analysis.

#### Biochemical Estimation

Blood was collected from retro-orbital route under mild anesthesia and allowed to clot and serum was separated by centrifugation at 3000 rpm for 10 min and stored in -80°C for further use. Serum biochemical enzymes like AST, ALT, ALP, and LDH levels was estimated by commercially available kits from Accurex, India as per the instructions given by the company. ELISA kits from R&D Systems, USA was used to measure the serum TNF-α, IL-1β, and IL-10 levels. Each sample was done in duplicate and results were expressed in Pg/ml.

#### Liver Biochemical Assays

Liver homogenate was prepared with 50 mM cold potassium phosphate buffer (pH 7.4). The resulting suspension was centrifuged at 3000 rpm for 15 min and supernatant was collected to measure the super oxide dismutase (SOD), catalase (CAT) by using assay kits from Cayman, USA as per the instructions given by the manufacturer and thiobarbituric acid reacting substances (TBARS) by Xia et al. (2013).

#### Histopathology Examination

Liver tissues were collected in 10% buffered formaldehyde from animals in different treatment groups and preserved at least for 24 h. After dehydration gradually with ethanol (70–100%), tissue was cleared in xylene and embedded in paraffin to make blocks. Sections (5 μm) was prepared by a Leica RM 2016 rotary microtome (Leica Instruments, Ltd., Shanghai, China) and stained with hematoxylin and eosin. Slides were examined under microscope (Leica Microsystems Digital Imaging, Germany) at 10x magnification to observe the histopathological changes (Bhardwaj et al., 2016; Choudhury A.J. et al., 2016).

### Effect of *A. reticulata* Fractions against Carrageenan Induced Paw Edema

Thirty six male adult wistar rats were randomly divided into six groups of six animals in each group. Animals kept overnight fasting with free access to water. Inflammation was induced in animals by injecting 0.05 ml of 1% carrageenan (Intraplantar route) to right hind paw. Following drug treatment was given to the animals 1 h prior to carrageenan injection:

**Group I:** Control animals (0.3% CMC orally).

**Group II:** Treatment with 10 mg/kg indomethacin orally.

**Groups III** **and** **IV:** Treatment with ARBME 200 and 400 mg/kg orally.

**Groups V** **and** **VI:** Treatment with ARBWF 50 and 100 mg/kg orally.

Paw volume was measured at 0, 1, 3, and 5 h after the induction of inflammation by water plethysmometer (Harvard apparatus, Panlab, Spain). After taking the readings, blood was collected to measure the serum inflammatory markers and all the animals were sacrificed.

#### Measurement of Inflammatory Cytokine Levels

Blood was centrifuged at 1000 rpm for 10 min to separate the serum. Serum inflammatory cytokines like TNF-α, Il-1β, and IL-10 levels were measured using commercially available kits from R&D Systems, USA by following manufactures guidelines. All the experiments were carried out at 4°C and results were presented in pg/ml.

### Statistical Analysis

All the results were expressed as mean ± SD. One way ANOVA followed by Tukey’s multiple comparison tests was used to compare the different parameters between the groups. A *P*-value < 0.05 was considered as significant. Graphpad prism 6 software was used to perform the statistical analysis.

## Results and Discussion

### *In Vitro* Anti-oxidant Activity of *A. reticulata* Fractions

#### DPPH Assay

**FIGURE 2:**
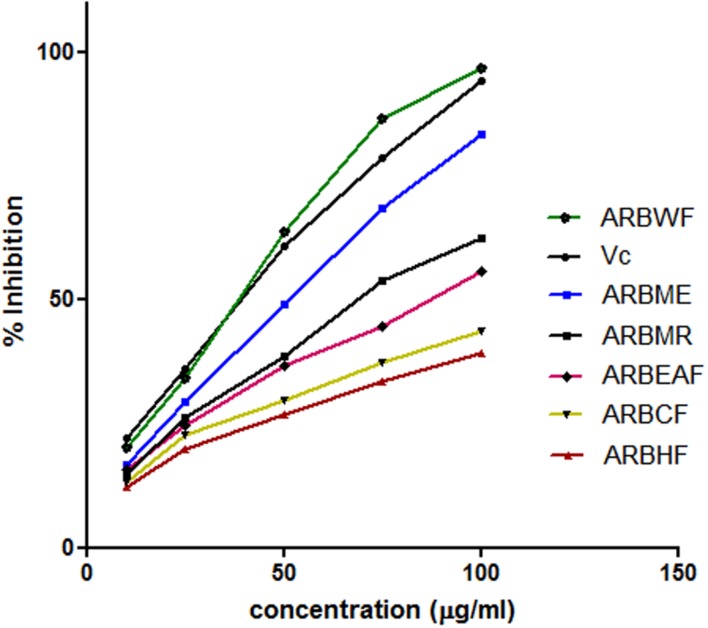
**Diphenyl picryl hydrazine (DPPH) radical scavenging activity of different fractions of *A. reticulata* bark**. ARBME, *A. reticulata* bark methanol extract; ARBHF, *A. reticulata* bark hexane fraction; ARBCF, *A. reticulata* bark chloroform fraction; ARBEAF, *A. reticulata* bark ethyl acetate fraction; ARBWF, *A. reticulata* bark water fraction; ARBMR, *A. reticulata* bark methanol residue; Vc, ascorbic acid. All the results were expressed in mean ± SD (*n* = 3).

This assay is based on the ability of the substance to reduce the stable DPPH free radical to DPPH. Scavenging ability of the antioxidant substance on DPPH is widely accepted method (Liu et al., 2013). The scavenging ability of the *A. reticulata* extract and fractions were given in **Figure 2**. All the fractions exhibited dose dependent radical scavenging ability. Among all the fractions ARBWF showed maximum response and ARBHF showed the lowest response. From these findings it is suggested that both ARBME and ARBWF have strong antioxidant property.

#### Reducing Power Assay

**FIGURE 3:**
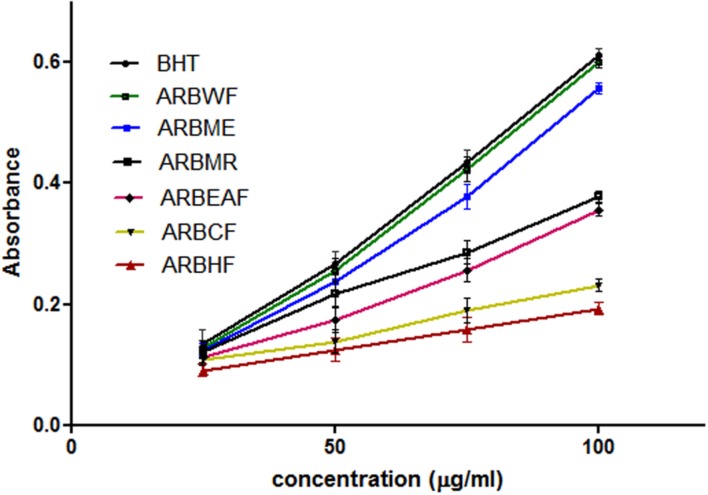
**Reducing power ability of different fractions of *A. reticulata* bark**. ARBME, *A. reticulata* bark methanol extract; ARBHF, *A. reticulata* bark hexane fraction; ARBCF, *A. reticulata* bark chloroform fraction; ARBEAF, *A. reticulata* bark ethyl acetate fraction; ARBWF, *A. reticulata* bark water fraction; ARBMR, *A. reticulata* bark methanol residue; BHT, butylated hydroxyl toluene. All the results were expressed in mean ± SD (*n* = 3).

Reducing power of the substance directly related with its antioxidant ability (Meir et al., 1995). To measure the reducing ability, transformation of Fe^3+^ to Fe^2+^ was taken into the consideration, which can be measured spectrophotometrically. Reducing power ability of *A. reticulata* extract and fractions were increased directly proportional to the concentration (**Figure 3**). ARBME and ARBWF showed promising results, where water fraction showed potent response. Among all the fractions tested ARBME and ARBWF showed potent renounce, so we further evaluated the biological activity of only these two fractions.

#### Total Antioxidant Activity

Photo-chemi-luminescence method in the Photochem instrument was dependent on the antioxidant concentration based on the Guldberg-Waage law, which expresses the magnitude of the reaction. Reactions of radicals with antioxidants give relatively stable products which are measurable. Antioxidant capacity of the drug/substance depends on the concentration (Hic and Balic, 2012). Total antioxidant activity of methanol extract and water fraction of *A. reticulata* bark found to be 57.34 nmol trolax equivalents and 87.16 nmol ascorbic acid equivalents respectively.

#### Cytotoxicity Effect of ARBME and ARBWF in HepG2 Cells

Both ARBME and ARBWF did not show significant difference on percentage growth inhibition up to 250 μg/ml. At large dose 500 μg/ml both the fractions showed growth inhibits the HepG2 cell growth, so we selected 50, 100, and 200 μg/ml to evaluate the hepato protective response of ARBME and ARBWF.

#### Cytoprotective Effect of ARBME and ARBWF in CCl_4_ Induced Toxicity on HepG2 Cells

**Table 1** shows the effect of different drug treatments on CCl_4_ intoxicated HepG2 cell lines. On exposure to CCl_4_ cell viability was significantly decreased and LDH levels were significantly raised. Cells on treatment with ARBME and ARBWF showed protection from CCl_4_ dose dependently which can be confirmed by increased in cell viability and decreased LDH levels. HepG2 cell model is widely used model to study hepatoprotective activity of xenobiotics, because of its resemblance to human hepatocytes (Krithikaa et al., 2009). The extent of damage to cells on exposure to CCl4 was determined by measuring the cell leakage enzyme like LDH. Here in this study LDH levels were significantly decrease and cell viability increased in ARBME and ARBWF treated groups compared to CCl4 alone treated group. This *in vitro* result explains the hepatoprotective activity of the ARBME and ARBWF, water fraction of methanol extract showed potent activity than methanol extract. Both ARBME and ARBWF was found to be non-toxic toward HepG2 cells, we further evaluated its activity in *in vivo* models.

**Table 1:** Protective effect of ARBME and ARBWF against CCl_4_ intoxicated HepG2 cell lines.

Group no.		Treatment	% Cell viability	LDH (U/L)
1	Group-I	Normal control	99.42 ± 0.26	96.64 ± 1.67
		DMSO control (0.25% V/V)	98.19 ± 0.18	95.47 ± 1.45
		Silymarin control (200 μg/ml)	98.56 ± 0.21	97.18 ± 1.58
		ARBME (200 μg/ml) control	98.07 ± 0.19	97.73 ± 1.76
		ARBWF (200 μg/ml) control	97.84 ± 0.22	96.29 ± 1.41
2	Group-II	Toxin CCl4 control (1% v/v)	19.72 ± 0.26^∧^	197.64 ± 3.38
3	Group-III	Silymarin 50 μg/ml + CCl_4_ (1% v/v)	68.22 ± 0.21^∗^	123.37 ± 2.19^∗^
		Silymarin 100 μg/ml + CCl_4_ (1% v/v)	76.47 ± 0.18^∗^	115.42 ± 2.35^∗^
		Silymarin 200 μg/ml + CCl_4_ (1% v/v)	88.39 ± 0.24^∗^	106.42 ± 1.84^∗^
4	Group-IV	ARBME 50 μg/ml + CCl_4_ (1% v/v)	39.64 ± 1.20^∗^	182.71 ± 2.46^∗^
		ARBME 100 μg/ml + CCl_4_ (1% v/v)	45.27 ± 1.23^∗^	159.65 ± 2.21^∗^
		ARBME 200 μg/ml + CCl_4_ (1% v/v)	52.66 ± 1.17^∗^	142.56 ± 1.87^∗^
5	Group-V	ARBWF 50 μg/ml + CCl_4_ (1% v/v)	47.25 ± 1.26^∗^	151.19 ± 1.49^∗^
		ARBWF 100 μg/ml + CCl_4_ (1% v/v)	56.17 ± 1.21^∗^	138.24 ± 2.17^∗^
		ARBWF 200 μg/ml + CCl_4_ (1% v/v)	64.42 ± 0.95^∗^	129.68 ± 1.41^∗^

*All the results were expressed in mean ± SD (*n* = 3). ^∧^*P* < 0.05 in comparison of group-II with group-I. ^∗^*P* < 0.05 in comparison of groups-III, IV, and V with group-II. ARBME, *A. reticulata* bark methanol extract; ARBWF, *A. reticulata* bark water fraction*.

#### Acute Toxicity Studies

ARBME and ARBWF at 2000 mg/kg did not show any effect on respiratory rate, heart rate, body temperature, salivation, corneal reflex, locomotor activity, body tone, skin tone, grip strength, abdominal tone, tremors, piloerection, tail elevation, twitches, and convulsions are also not observed. No mortality was observed after 14 days of observation period in tested mice. ARBME and ARBWF were found to be non-toxic to animals, so we further evaluated the hepatoprotective and anti-inflammatory activity of same using appropriate animal models.

### Hepatoprotective Activity of ARBME and ARBWF against CCl_4_ Induced Hepatic Damage

#### Effect of Drug Treatment on Serum Hepatobiliary Enzyme Levels

Hepato toxins cause damage to the plasma membrane of hepatocytes and cause liver toxicity. CCl_4_ is well-known hepatotoxic to induce liver damage in laboratory animals. Active metabolite (CCl_3•_) is mainly associated with hepatotoxicity induced by CCl_4_. This (CCl_3•_) radical covalently binds with sulfhydryl groups of protein thiols in hepatocytes and cause lipid peroxidation and necrosis. During this necrotic stage hepatobiliary enzymes releases into blood circulation, which shows increase levels in serum (Vuda et al., 2012). CCl_4_ alone treated animals showed raised levels of all serum biochemical markers. Standard drug silymarin, ARBME and ARBWF restored the elevated levels of serum AST, ALT, ALP, and LDH levels due to CCl_4_ toxicity. ARDWF showed more potent activity than ARBME, but the response is not up to standard drug activity. The results of hepatoprotective activity of ARBME and ARBWF were summarized in **Table 2**.

**Table 2:** Effect of different drug treatment on serum and liver enzyme levels of CCl_4_ intoxicated rats.

Group no.	Group	Serum levels of				Liver levels of		
		AST (IU/l)	ALT (IU/l)	ALP (IU/l)	LDH (U/l)	SOD (U/ml)	CAT (nmol/min/ml)	TBARS (nmol/g tissue)
1	Control	41.6 ± 2.8	38.3 ± 2.4	94.5 ± 3.2	436.8 ± 15.4	9.8 ± 1.1	5.2 ± 0.8	158.5 ± 8.7
2	CCl_4_ treatment (1.5 ml/kg)	124.2 ± 6.1^$$$^	96.7 ± 5.8^$$$^	197.5 ± 9.7^$$$^	1022.9 ± 29.2^$$$^	1.9 ± 0.4^$$$^	0.8 ± 0.1^$$$^	342.8 ± 12.6^$$$^
3	Silymarin (100 mg/kg) + CCl_4_ (1.5 ml/kg)	52.8 ± 4.4^∗∗∗^	44.6 ± 3.5^∗∗∗^	108.2 ± 7.8^∗∗∗^	471.4 ± 20.6^∗∗∗^	7.7 ± 0.8^∗∗∗^	4.6 ± 0.5^∗∗∗^	171.8 ± 9.4^∗∗∗^
4	ARBME 200 mg/kg + CCl_4_ (1.5 ml/kg)	72.7 ± 5.3^∗∗∗^	59.9 ± 3.8^∗∗∗^	132.4 ± 7.2^∗∗∗^	563.8 ± 18.5^∗∗∗^	3.8 ± 0.7^∗∗∗^	3.2 ± 0.4^∗∗^	213.4 ± 11.8^∗∗∗^
5	ARBME 400 mg/kg + CCl_4_ (1.5 ml/kg)	63.4 ± 4.8^∗∗∗^	53.4 ± 4.1^∗∗∗^	121.8 ± 9.6^∗∗∗^	509.4 ± 16.6^∗∗∗^	5.6 ± 1.1^∗∗∗^	3.9 ± 0.6^∗∗∗^	186.1 ± 8.6^∗∗∗^
6	ARBWF 50 mg/kg + CCl_4_ (1.5 ml/kg)	67.6 ± 5.4^∗∗∗^	53.9 ± 4.7^∗∗∗^	126.9 ± 8.5^∗∗∗^	530.7 ± 19.2^∗∗∗^	5.1 ± 0.9^∗∗∗^	3.8 ± 0.4^∗∗∗^	194.5 ± 9.7^∗∗∗^
7	ARBWF 100 mg/kg + CCl_4_ (1.5 ml/kg)	57.3 ± 3.9^∗∗∗^	45.1 ± 3.6^∗∗∗^	111.2 ± 8.8^∗∗∗^	494.3 ± 21.2^∗∗∗^	6.8 ± 0.9^∗∗∗^	4.5 ± 0.7^∗∗∗^	178.6 ± 10.5^∗∗∗^

*All the results were expressed in mean ± SD (*n* = 6). $$$*P* < 0.001 in comparison of CCl_4_ alone treated animals with normal animals. ^∗∗∗^*P* < 0.001 and ^∗∗^*P* < 0.01 in comparison of drug treated animals with CCl_4_ alone treated animals. ARBME, *A. reticulata* bark methanol extract; ARBWF, *A. reticulata* bark water fraction*.

#### Effect of Drug Treatment on Lipid Peroxidation and Liver Anti-Oxidant Enzymes

Thiobarbituric acid reacting substances are the end products of lipid peroxidation, high levels of these substances inside the body is a marker to liver injury. SOD and catalase are the antioxidant defense enzymes that protect liver from free radicals and inhibit the lipid peroxidation. CCl_4_ treatment cause significant decrease in these antioxidant enzymes and cause the lipid peroxidation (Cheng et al., 2013). Treatment with standard drug silymarin, ARBME and ARBWF significantly increases the levels of SOD and catalase and inhibits the lipid peroxidation (**Table 2**). Results of this study clearly demonstrate the hepatoprotective activity of ARBME and ARBWF. The water fraction (ARBWF) showed potent response than methanol extract (ARBME), which states that polar compounds of *A. reticulata* bark was responsible for the hepatoprotective response.

#### Effect of Drug Treatment on Cytokine Levels

**FIGURE 4:**
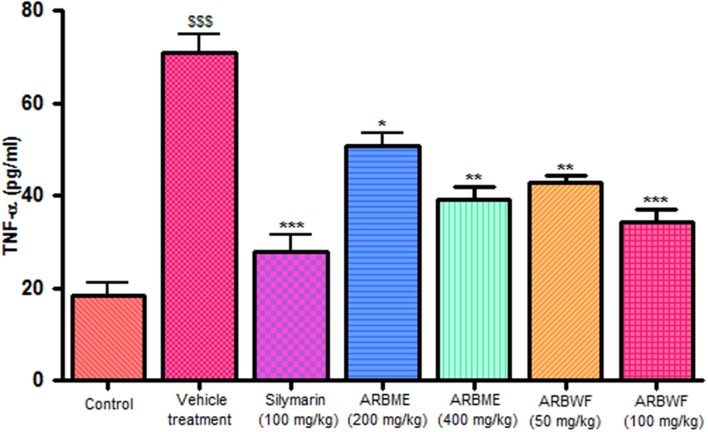
**Effect of different drug treatment on serum TNF-α levels**. All the results were expressed in mean ± SD (*n* = 6). ^$$$^*P* < 0.001 in comparison of CCl_4_ alone treated animals with normal animals. ^∗∗∗^*P* < 0.001, ^∗∗^*P* < 0.01, and ^∗^*P* < 0.05 in comparison of drug treated animals with CCl_4_ alone treated animals. ARBME, *A. reticulata* bark methanol extract; ARBWF, *A. reticulata* bark water fraction.

**FIGURE 5:**
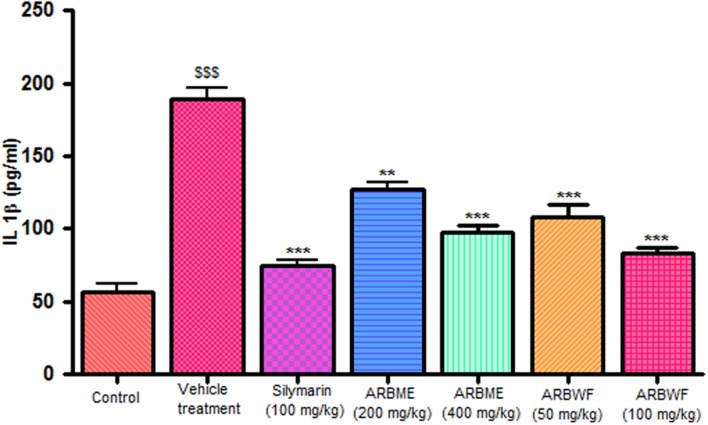
**Effect of different drug treatment on serum IL-1β levels**. All the results were expressed in mean ± SD (*n* = 6). ^$$$^*P* < 0.001 in comparison of CCl_4_ alone treated animals with normal animals. ^∗∗∗^*P* < 0.001 and ^∗∗^*P* < 0.01 in comparison of drug treated animals with CCl_4_ alone treated animals. ARBME, *A. reticulata* bark methanol extract; ARBWF, *A. reticulata* bark water fraction.

**FIGURE 6:**
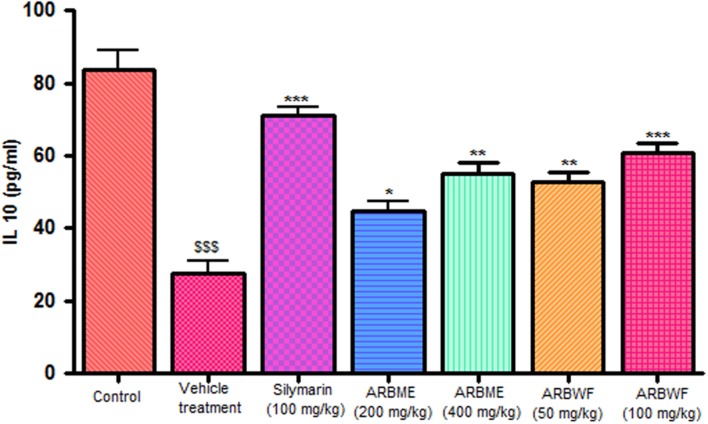
**Effect of different drug treatment on serum IL-10 levels**. All the results were expressed in mean ± SD (*n* = 6). ^$$$^*P* < 0.001 in comparison of CCl_4_ alone treated animals with normal animals. ^∗∗∗^*P* < 0.001, ^∗∗^*P* < 0.01, and ^∗^*P* < 0.05 in comparison of drug treated animals with CCl_4_ alone treated animals. ARBME, *A. reticulata* bark methanol extract; ARBWF, *A. reticulata* bark water fraction.

CCl_4_ induction activates inflammatory cytokines TNF-α, IL-1β and decrease anti-inflammatory cytokine IL-10 levels in *in vivo* system. CCl_4_ metabolism stimulated the Kupffer cells which activates this TNF-α and IL-1β. IL-1β is strong inflammatory cytokines which involves in the production of prostaglandins, macrophage activation and neutrophil infiltration. This activation of inflammatory cytokine cascade produces significant damage to the liver cells (Edwards et al., 1993; Ishiyama et al., 1995). Pretreatment with standard drug silymarin, ARBME and ARBWF before CCl_4_ induction significantly inhibits the TNF-α (**Figure 4**), IL-1β (**Figure 5**) production and IL-10 (**Figure 6**) destruction in animals. ARBWF showed potent response to ARBMF which confirms that polar compounds of ARB possess anti-inflammatory activity mediated hepatoprotective response.

#### Effect of Drug Treatment on Histopathology of CCl_4_ Intoxicated Rat Liver

**FIGURE 7:**
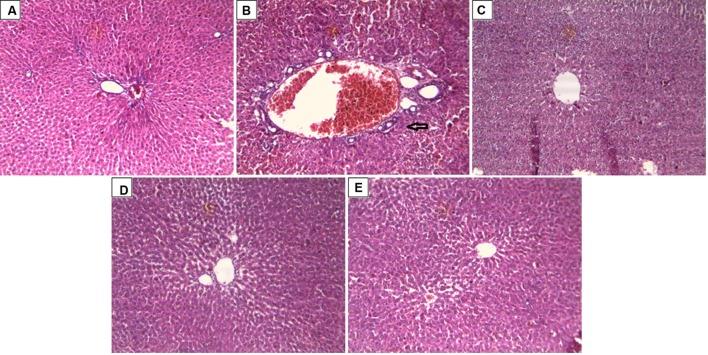
**Effect of drug treatment on liver histopathology of CCl_4_ intoxicated rats**. **(A)** Liver of control rat with no CCl_4_ treatment showing normal hepatocytes with no inflammatory cell infiltrate; x10. **(B)** Liver of rat treated with CCl4 showing formation of bridging necrosis with chronic inflammatory cells; x10. **(C)** Liver of rat treated with CCl4 and 100 mg/kg silymarin showing normal hepatocytes with no inflammation and necrosis; x10. **(D)** Liver of rat treated with CCl4 and 400 mg/kg of ARBME showing mild periportal inflammation and no necrosis; x10. **(E)** Liver of rat treated with CCl4 and 100 mg/kg ARBWF showing absence of inflammatory cells or necrosis; x10. ARBME, *A. reticulata* bark methanol extract; ARBWF, *A. reticulata* bark water fraction.

In support to the hepatoprotective activity of ARBME and ARBWF, histopathology of liver tissue was performed and analyzed. Liver tissue of control animals showed normal hepatocytes with no inflammatory cell infiltrate (**Figure 7A**). CCl_4_ intoxication causes demolishment of hepatocytes which was evidenced by formation of bridging necrosis, collagen accumulation, large septa and chronic inflammation (**Figure 7B**). Standard drug silymarin treated group, ARBME (400 mg/kg) and ARBWF (100 mg/kg) pretreated animals showed significant protection from the CCl_4_, where normal tissue architecture with no necrosis and mild inflammation was observed (**Figures 7C–E**). From this evidence the present study confirms the hepatoprotective ability of ARB. Compared to ARBME the dose of ARBWF is less which states that, the later one is potent than earlier.

### Effect of Drug Treatment on Carrageenan Induced Paw Edema

#### Paw Volume

**FIGURE 8:**
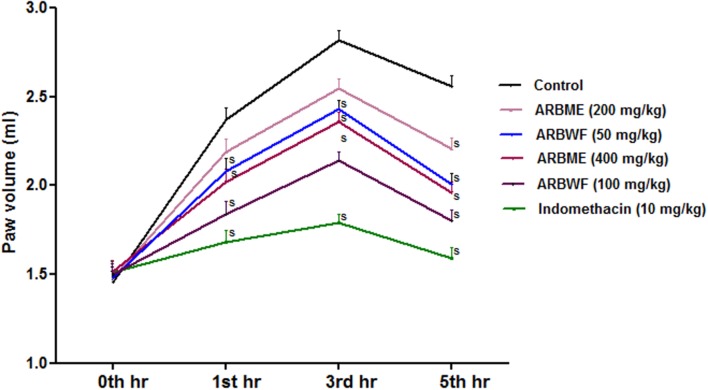
**Effect of *A. reticulata* bark fractions on carrageenan induced paw edema over 5 h**. All the results were expressed in mean ± SD (*n* = 6). S *P* < 0.05 in comparison of drug treated animals with saline treated animals. ARBME, *A. reticulata* bark methanol extract; ARBWF, *A. reticulata* bark water fraction.

Carrageenan induced inflammation in experimental animals is the widely used model to test the orally injected anti-inflammatory agents/drugs. Inflammation process takes place in two phases, in initial phase histamine and serotonin releases and second phase involves the activation of prostaglandins and lysosomal bodies which are the prime target of most of anti-inflammatory agents (Marroquin-Segura et al., 2009). Standard drug indomethacin, ARBME and ARBWF significantly decrease the carrageenan induced paw edema in rats (**Figure 8**) Further ARBWF showed potent response to ARBME which demonstrate that polar chemical components of ARB were responsible for this action.

#### Serum Cytokine Levels

**FIGURE 9:**
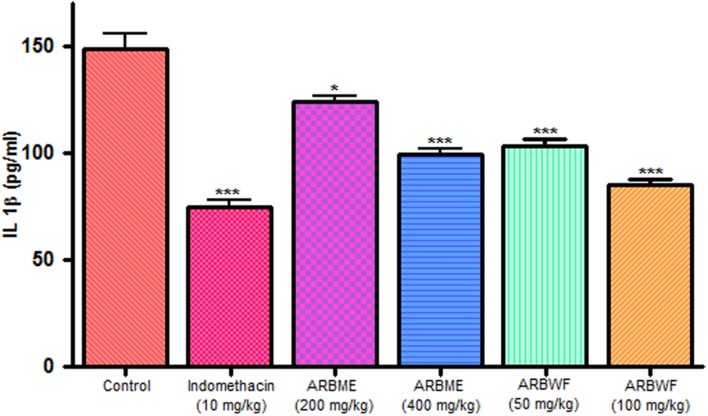
**Effect of different drug treatment on serum IL-1β levels of carrageenan induced paw edema rats**. All the results were expressed in mean ± SD (*n* = 6). ^∗∗∗^*P* < 0.001 and ^∗^*P* < 0.05 in comparison of drug treated animals with saline treated animals. ARBME, *A. reticulata* bark methanol extract; ARBWF, *A. reticulata* bark water fraction.

**FIGURE 10:**
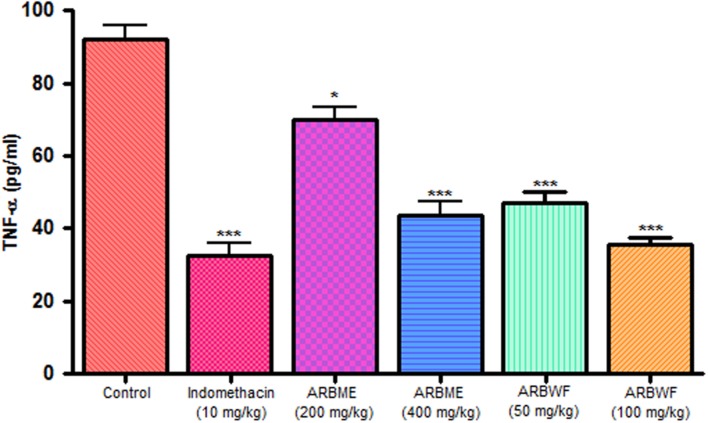
**Effect of different drug treatment on serum TNF-α levels of carrageenan induced paw edema rats**. All the results were expressed in mean ± SD (*n* = 6). ^∗∗∗^*P* < 0.001 and ^∗^*P* < 0.05 in comparison of drug treated animals with saline treated animals. ARBME, *A. reticulata* bark methanol extract; ARBWF, *A. reticulata* bark water fraction.

As discussed previously pro inflammatory cytokines like IL-1β and TNF-α plays a major role in inflammation process through activation of the prostaglandin and macrophages. Carrageenan injection cause notable rise in IL-1β and TNF-α levels and standard drug indomethacin, ARBME and ARBWF treated animals inhibits the production of these cytokines (**Figures 9** and **10**). Our findings demonstrate the anti-inflammatory ability of ARB.

## Conclusion

The present study demonstrates the antioxidant, hepatoprotective and anti- inflammatory ability of bioactive guided fractions of *A. reticulata* bark. Methanol extract of ARB and its water fraction showed strong antioxidant activity and hence further evaluated for biological activity. Both the fractions exhibited significant hepatoprotection and anti-inflammatory response in both *in vitro* and *in vivo* studies. The phyto constituents of ARB potentially reduced the oxidative stress and inflammatory cytokines to yield the therapeutic response. ARBWF showed potent response in all the tests conducted, so from this study it can be concluded that polar chemical components of *A. reticulata* bark like 16-a-hydroxy-(e)-kauran-19-oic acid, diterpenes (e)-kaur-16-en-19-oic acid, reticullacinone, methyl-17-hydroxy-16-b-(e)-kauran-19-oate, rolliniastatin and molvizarin might be responsible for the obtained pharmacological activity. ARBWF can be used to prepare the herbal formulations for the treatment of liver aliments and inflammation related diseases. Successful isolation and biological screening of the chemical component responsible for the activity can potentially contribute toward development of novel drug entity which is undergoing in our laboratory.

## Author Contributions

Mr. RK designed the whole study and performed all the *in vitro* and *in vivo* experiments and wrote the manuscript. Mr. SK, Miss. BC, and Mr. SM helped Mr. RK in all the experiments conducted and preparation of manuscript. Dr. KK performed and analyzed the histopathology of liver. Professor JK and Professor SD supervised the all experimental work and corrected the manuscript.

## Conflict of Interest Statement

The authors declare that the research was conducted in the absence of any commercial or financial relationships that could be construed as a potential conflict of interest.
